# Clinical application of pulse-gated non-enhanced rapid magnetic resonance imaging in the definitive diagnosis of aortic dissection

**DOI:** 10.1016/j.clinsp.2024.100467

**Published:** 2024-08-30

**Authors:** QinWen Yan, Gang Hu, Qin Wang, Lei Wu, Jun Zhang, Lan He, CiLai Jiao, Si Ma, MinChao Xiong

**Affiliations:** Department of Medical Imaging (Radiology), Ezhou Central Hospital Affiliated to Hubei University of Science and Technology, Ezhou City, Hubei Province, PR China

**Keywords:** Aortic dissection, Pulse-gating, Cardiac-gated, True fast imaging with steady-state precession (True-FISP), Half-Fourier acquisition single-shot turbo spin echo (HASTE), Magnetic resonance imaging

## Abstract

**Objective:**

Aortic Dissection (AD) is one of the most fatal acute diseases in cardiovascular diseases, with rapid onset and progression and a high fatality rate. This study aims to investigate the clinical values of non-enhancement peripheral pulse-gating rapid magnetic resonance imaging in deterministic diagnosis of AD.

**Methods:**

Aorta magnetic resonance imaging was performed in 21 healthy volunteers at a 1.5t MR scanner sequences including cardiac-gated and peripheral pulse-gated True-FISP and HASTE were carried out separately. Acquisition Time (TA), Signal to Noise Ratio (SNR), Contrast Noise Ratio (CNR), and entirety of vessel wall blood flow artifacts were measured and compared. A total of 56 AD cases were displayed by non-enhancement peripheral pulse-gating fast MR imaging, and the results were compared with pathological findings or CTA of the aorta. The dissection rupture, tear film, true and false lumen, thrombosis, hydropericardium, and the main branches of AD were evaluated respectively.

**Results:**

There were no significant differences in SNR, CNR, entirety of the vessel wall, and blood flow artifact between cardiac-gated and peripheral pulse-gated fast MR imaging. Non-enhancement pulse-gated fast scanning takes less TA time. By the pulse-gated non-enhancement fast MR imaging, the dissection rupture, tear film, true and false cavity, thrombosis, hydropericardium, and the main branches of aortic dissection were shown clearly. Multi-planar and multi-angle scans helped to show the extent of entrapment rupture, whereas partial complex tears or bi-directional tears were slightly less well visualized.

**Conclusion:**

Non-enhancement peripheral pulse-gated rapid magnetic resonance imaging can be used for deterministic diagnosis of AD.

## Highlights

• Free breathing pulse gated non-enhanced rapid MR imaging can obtain images with diagnostic value.
• Multi-plane and multi-angle thin-layer scanning are favorable for the display of the rupture, while some complex tears and bidirectional tears are poorly displayed.
• Free breathing pulse gated non-enhanced rapid MR imaging technique can be used for the exclusive and definitive diagnosis of aortic dissection.
• Non-enhancement peripheral pulse-gating rapid magnetic resonance imaging can be used for deterministic diagnosis of aortic dissection.

## Introduction

Aortic Dissection (AD) is a disease in which the aortic lining is ruptured and blood enters the middle layer from the ruptured opening, separating the aortic wall and forming true and false lumen^1^ AD is one of the most fatal acute diseases in cardiovascular diseases, with rapid onset, rapid progression and high fatality rate. It has been reported that the sudden death rate at the time of onset is 3%, the mortality rate in 48-hour is 50%, and the mortality rate in 1-week is 70%‒80%.^2^, ^3^, ^4^, ^5^, ^6^ In recent years, the incidence of AD has been increasing year by year. Due to its complex and changeable condition and lack of specific symptoms and signs, the rate of misdiagnosis is high at grassroots hospitals, and medical disputes frequently occur due to improper treatment and delayed delivery. Therefore, rapid, non-invasive, accurate, and timely diagnosis is crucial for the treatment and prognosis of AD patients.

Transthoracic Echocardiography (TTE), Trans Esophageal Echocardiography (TEE). CT Angiography (CTA), and Contrast-Enhanced Magnetic Resonance Angiography (Ce-MRA) are methods of diagnosing AD at home and abroad.^7^, ^8^, ^9^ TTE and TEE can distinguish the true and false lumen of AD, display the internal diaphragm of the dissection, examine the cardiac structure and hemodynamics to help determine the etiology of AD, which can be conducted beside the bed with quick results, and they can be repeated conducted for examination on-site, as a result, it is suitable for rapid diagnosis of emergency patients. TTE shows that the function of distal ascending aorta and descending aorta is limited. Esophageal intubation is required in tee examination, which is not widely used in China.^10^ CTA sensitivity stands at 100% and specificity at 98%‒99%, and it can scan the whole aorta, including true and false lumen, the wall of blood vessels and the surrounding structure, Intramural Hematomas (IMH) and Penetrating Atherosclerotic Ulcer (PAU). The disadvantage is that the interlayer entrance cannot be explored and cardiac function cannot be evaluated.^11^^,^^12^ Enhanced sensitivity and specificity of MRA are equal to or even better than the TEE, CTA.^13^^,^^14^ With the development of high-magnetic field MR technology, MR has gradually substituted damaged aortic angiography as the gold standard for the diagnosis of AD.^15^ However, due to the limitations of cost, long imaging time and some contraindications, its clinical application is limited. Although aortic CTA and enhanced MRA have important advantages in determining the location of AD rupture, the extent of intervention, and the location and form of the true and false lumen. However, the examination is lengthy, invasive to the patient, and requires injection of contrast agent.^16^

At present, rapid and high-field MR imaging of AD is generally performed by ECG controlled breath-holding scanning technology. Some patients fail to tolerate and hold their breath for a long time due to severe chest pain, electrodes to the patient's body surface are required in ECG control, which is relatively complicated and time-consuming to operate, and the position of electrodes will affect the ECG waveform and thus affect the trigger.^17^ True FISP and HASTE sequences are among the most commonly used high-field MR fast imaging sequences. The former belongs to the gradient echo balanced steady-state free precession sequence, while the HASTE sequence belongs to the semi-Fourier acquisition single-excitation fast spin echo sequence, and the common characteristics of the two sequences are that they are not sensitive to respiratory movement artifacts.^17^ In order to eliminate the impact of cardiac macrovascular pulsation on the image, proton excitation, and signal acquisition can be triggered by gated technology in a fixed phase of cardiac macrovascular pulsation to eliminate the artifacts.^18^^,^^19^ The most commonly used gated techniques include ECG and pulse gating. The former adopts electrodes attached to the chest wall to obtain ECG signals with *R* wave as the trigger point for MR measurement, and selects appropriate delay time to make MR signal acquisition fixed at the same phase of the cardiac cycle. Peripheral pulse trigger is to use an optical pulse sensor, and according to the flow of blood pulsing to the whole body to trigger the MRI data. As for a specific scan, the pulse clamp is used to clamp the thumb, and the pulse wave troughs as an MR signal acquisition window. Although the trigger time of electrocardiogram control is slightly delayed, the stable pulse can also collect an image that meets the diagnostic needs. It has been reported in the literature that the combination of rapid spin echo sequence of plaque analysis and electrocardiogram control can improve the overall clarity of vascular wall image.^20^

In this study, the emergency examination process and imaging technique were improved in clinical practice according to the characteristics of MR ultrafast sequence imaging. Using pulse-gated control and free-breathing acquisition techniques, we attempted to perform the whole rapid imaging of the aorta to achieve the effect of completely non-invasive, no contrast agent, and no breath-holding. In addition, the whole examination time is controlled within 5‒7 minutes, with the inhalation and exhalation time not exceeding 10 minutes, so as to make a clear and exclusive diagnosis of AD and provide a reference for timely clinical treatment and prognosis judgment.

## Materials and methods

This study followed the STROBE statement.

### Clinical data

Pulse-gated True-FISP rapid imaging (True-FISP and HASTE Sequence, Peripheral pulse-gated): Twenty-one healthy volunteers, including 14 males and 7 females, were aged 27‒91 years with a mean age of (63.4 ± 13.2) years.

True-FISP rapid imaging (True-FISP and HASTE Sequence, Cardiac-gated): Twenty-one healthy volunteers, including 12 males and 9 females, ranged in age from 44‒85 years with a mean age of (64.8 ± 10.3) years.

There were 56 patients with AD confirmed by surgical pathology and aortic CTA, including 41 males and 15 females, aged from 35‒83 years, with an average age of 54.7 ± 11.5 years. The mean time from diagnosis to MIR in patients with dissection was 4.6 days.

### MR imaging system

A Siemens magnetom essenza 1.5t superconducting magnetic resonance imager was used with a gradient field strength of 45 mt/m, a cutting speed of 200 mt/(mm.s), a body surface phased array coil software version syngo MR c13, and pulse-gate and ECG-gate scanning techniques.

### Scan sequence and imaging parameters

The specific scanning parameters are as follows: True-FISP (bright blood) sequence, TR: 306.91∼427 ms, TE: 1.5∼1.6 ms, FOV: 295 × 360 mm, layer thickness: 6.5 mm, matrix 124 × 256, Flip Angle (FA): 80°, excitation times (average) 1-time, acceleration factor (echo chain etl): 2, Bandwidth (BW): 890 hz/px, Echo gap (ES) 3.6 ms; HASTE (black blood) sequence, TR: 750 ms, TE: 41 ms, FOV: 295×360 mm, layer thickness: 6.5 mm, matrix 124×256, Turning Angle (FA): 160°, excitation times (average) 1-time, acceleration factor (echo chain etl): 126, Bandwidth (BW): 789 hz/px, Echo gap (ES) 4.12 ms. The scan was conducted from the ascending aortic root to the bifurcation of the common iliac artery. Three scan areas were normally divided into three scan areas, and there was partial overlap between adjacent areas during positioning. Finally, the oblique sagittal position of the thoracic aorta was completed with scanning. During the examination, the patient's heart rate changes will affect the tr value and scanning time of the bright blood sequence.

### Measurement index and calculation method

Acquisition Time (TA) of True-FISP (bright blood) sequence and HASTE (black blood) sequence were recorded under two gating methods.

Measuring gating pulse and heart switch control True-FISP bright blood sequence image SNR, CNR, measurement work was done in siemens magnetom essenza 1.5t which serves as superconducting magnetic resonance imaging workstation, each sequence was measured in the same anatomical level and same area selected area (ROI), all the volunteers’ images were all measured by choosing dry plane of the pulmonary artery. The SD background was the standard deviation of the background noise (Standard Deviation [SD], the SI muscle was the Signal Intensity [SI] of the measured level homogeneous muscle, and the SI aorta was the signal intensity of the aortic cavity at the same level. Snr=SImuscle/SDbackground;CNR=SIaorta−SImuscle/SDbackground.

The blood flow artifact of target blood vessels was evaluated, as well as the cross-section artifact of the black blood sequence. Evaluation criteria: The vascular lumen signal is low and homogeneous, which means that there is no blood flow artifact. The signal in the vascular lumen is not heterogeneous, and abnormal interference signals such as small flake, line and vortex are visible to the eyes, which signifies blood flow artifacts.

With the results of surgical pathology and aortic CTA as the control, 56 cases of AD were classified with reference to the clinical Stanford classification standard, and the imaging manifestations of all types of AD were evaluated, including rupture, true and false lumen, thrombosis, main branch vascular involvement, pericardial effusion, etc.

SPSS 11.0 statistical software package was used for statistical analysis. The mean value test of the two samples was used for measurement data, and the count data was analyzed using the χ^2^ test; p < 0.05 was statistically significant.

## Results

**Fig. 1:**
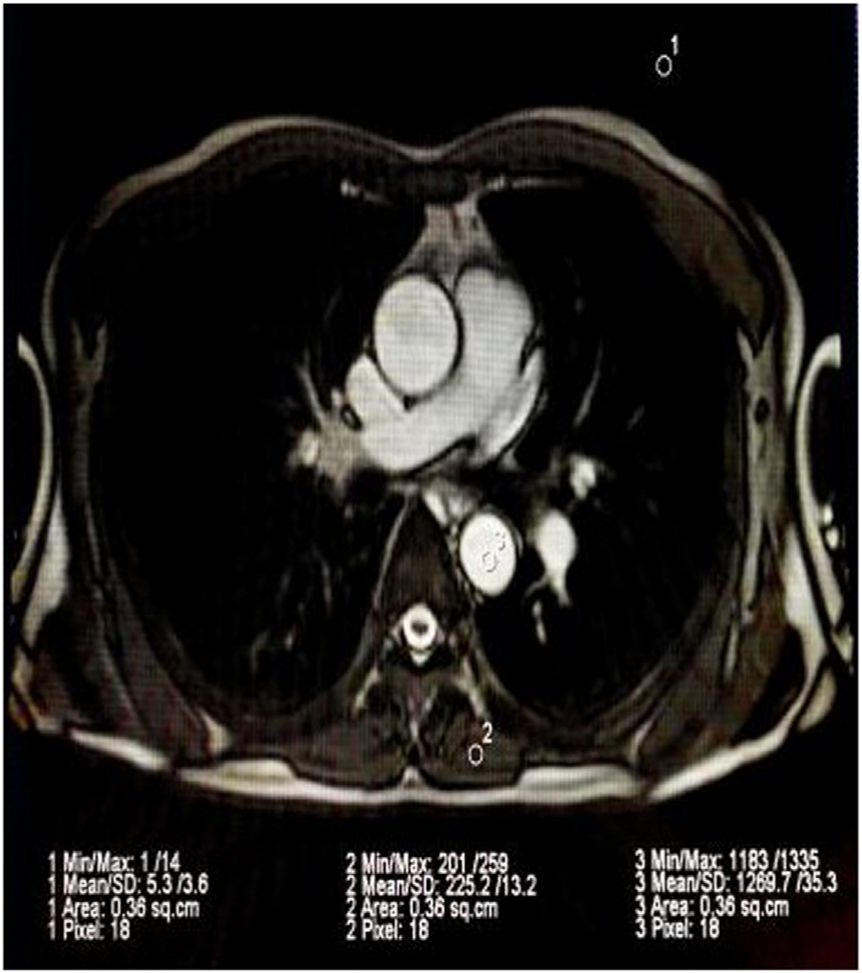
CNR image, SNR measurement.

**Fig. 2:**
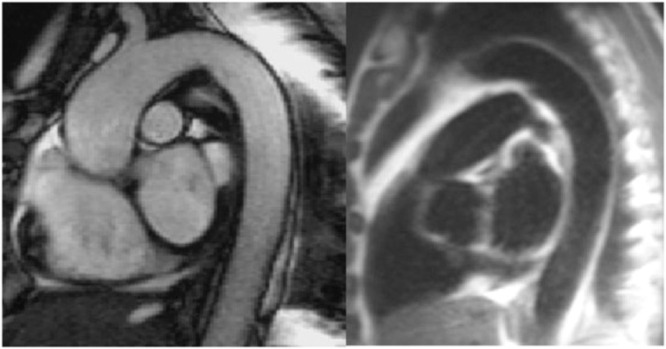
True-FISP bright blood sequence and HASTE black blood sequence: sagittal normal arch of the aorta and its descending part.

The whole process of the aorta was scanned using True-FISP and HASTE sequences, respectively. 21 volunteers and 21 volunteers were tested by using the non-breath-holding pulse gating method. The CNR and SNR of the bright blood sequence images of the two gated methods are shown in Table 1. According to the *t*-test of the mean values of the two samples, the difference between them was not statistically significant (p > 0.05). The presence of blood flow artifacts in the black blood sequence images of the two gated methods was shown in Table 2, and the difference between the two was not statistically significant (p > 0.05). The comparison of the scanning time between the non-breath-holding pulse-gated method and the breath-holding electrovalve method is shown in Table 3. The difference between the two samples was statistically significant (p < 0.005) by means of *t*-test. The scanning images are shown in Fig. 1, Fig. 2

**Table 1:** Comparison of CNR and SNR of bright blood sequence images of ECG and pulse-gated True-FISP.

Measurement index	ECG-gating	The pulse of the gating	*t*-value	*V*	p-value
CNR	295.01 ± 28.50	302.40 ± 28.14	0.846	40	0.4 < p < 0.5
SNR	70.40 ± 8.42	76.37 ± 12.51	1.810	40	0.05 < p < 0.1

**Table 2:** Comparison of blood flow artifacts between ECG gating control and pulse gated HASTE black blood sequence.

Gated mode	With artifacts	Without artifacts	*T*-total	χ^2^ value	p-value
The pulse of the gating	4	17	21		
ECG-gating	5	16	21		
Total	9	33	42	0.141	p > 0.5

**Table 3:** Comparison of scanning time of ECG and pulse gating.

Measurement index	ECG-gating	Pulse gating	*t*-value	*V*	p-value
Time (min)	9.58 ± 0.41	6.67 ± 0.53	26.45	45	p < 0.005

Among the 56 cases of AD confirmed by surgical pathology and aortic CTA, there were 19 cases with Stanford type-a and 33 cases with Stanford type-b, and 4 cases with atypical dissection that could not be classified as both, which are mainly presented with the symptom of AD located in the abdominal aorta with limited lesion range. Among them, 16 cases of type-a rupture were shown, 28 cases of type-b rupture were shown, and all 4 cases of atypical, intercalated rupture were shown, with the rupture display rates of 84.2%, 84.8%, and 100%, and the true and false lumen rates stood at 100%, and thrombosis and pericardial effusion were clearly shown, as shown in Table 4. There was a difference between Stanford type-a and Stanford type-b in 56 cases with AD. The former mainly involves the brachiocephalic artery, the left common carotid artery, and the left subclavian artery. The latter were mainly affected by the celiac trunk, the superior mesenteric artery, the renal artery, and the common iliac artery, respectively. The type-b dissection with reverse tear may also involve the three branches of the aortic arch, as shown in Table 5. The scanning images are shown in Supplementary Figs. 1‒9.

**Table 4:** Manifestations of aortic dissection rupture, true and false lumen, thrombosis and pericardial effusion in 56 cases.

Type	Cases	Crevasse display	True and false lumen	Thrombogenesis	Hydropericardium	Re-opening form
Stanford A	19	16	19	9	11	4
Stanford B	33	28	33	13	9	5
Atypical interlayer	4	4	4	1	0	0

**Table 5:** Branch vascular involvement of 56 cases of aortic dissection.

Type	Cases	Brachioc-ephalic artery	Left common carotid artery	Left subclavian artery	Coeliac trunk	Superior mesenteric artery	Left pulmo-nary artery	The right renal artery	Left common iliac artery	Right common iliac artery
Stanford A	19	11	10	8	7	7	9	8	11	10
Stanford B	33	3	5	6	24	21	19	17	18	16
Atypical interlayer	4	0	0	0	1	2	0	0	0	0

## Discussion

AD is one of the most common acute aortic syndromes, characterized by a dangerous course, complex etiology, and multiple etiologic interactions. AD has a rapid onset and a high rate of clinical misdiagnosis and underdiagnosis, making early diagnosis of the syndrome of great significance.^21^

In recent years, the high field MR imaging in the clinical application of big blood vessels than gaining fast imaging has gained popularity and plays an important role in the emergency exemplary diagnosis of aortic lesions (such as aortic dissection aneurysm, intramural hematoma, penetrating ulcer, etc.), especially for older, patients in a critical condition, those allergic to contrast agent or those with potential allergic reactions. Among them, pulse-gated non-enhanced rapid MR imaging can improve the temporal resolution of the examination, and patients can complete the examination in the state of free breathing without holding their breath, which greatly simplifies the examination process.^22^^,^^23^ In the present study, rapid image acquisitions of Peripheral pulse-gated and Cardiac-gated were compared under cardiac control, breath-holding, and pulse-gated free-breathing conditions using True-FISP and HASTE rapid magnetic resonance imaging sequences. The results showed that there was no statistical difference in SNR and CNR between the two methods, and both of them could obtain MR images with diagnostic value compared with electrocardiogram control, pulse gate control is easy to use with low cost and is beneficial to shorten the time of patient examination. Therefore, the authors believe that in the exclusive and deterministic diagnosis of emergency aortic dissection, pulse gate control can be used instead of electrocardiogram control, which can greatly shorten the examination time and simplify the examination process. The examination can be completed within 10 minutes for most patients.

For patients with emergency CTA, enhanced MRA examination, or TTE and TEE examination that cannot be definitively diagnosed, this study provides them with another possible option. With the clinical application of a cardiac pacemaker that can work normally in the high-field environment, the examination of absolute contraindications only requires the exclusion of metal foreign body in the body and the patients with early pregnancy within 3 months, and the examination is not affected by orthopedic internal fixation implants and various anastomosis devices.^24^ The 56 cases of aortic dissection confirmed by surgery pathology results and CTA in ruptures, torn internal diaphragm, the true and false lumen, thrombosis, pericardial effusion and main branch involvement were comprehensively analyzed. The results show that the free respiration gating pulse non-enhanced fast MR imaging can fully achieve the purpose of diagnosis or assertive analysis. CTA is obviously better than the display of crevasse, the impact of poor quality of imaging on diagnosis caused by motion artifact can be made up by using multi-plane and multi-angle scanning technology. Due to the timely diagnosis, most of the patients were treated quickly and effectively, so as to avoid the medical disputes caused by the untimely diagnosis, inaccuracy and underestimation of the disease. In a large number of screening cases in outpatient and emergency, in addition to aortic dissection, many common aortic lesions were also diagnosed with suggestive or definitive diagnoses. Such as atherosclerotic plaque. true pseudoaneurysm, interaortic hematoma, aortic perforative ulcer etc., which may also be one of the exploratory directions of this study.

In conclusion, this study explored the feasibility of rapid noninvasive Magnetic Resonance Imaging (MRIS) in the clinical application of deterministic diagnosis of aortic dissection and achieved the expected purpose. Through the optimization of the examination process and scanning technology, the examination time for patients is shortened to 7 minutes and the passage time is limited to 10 minutes, and answers to several major questions of clinical concern are as follows: 1) Is there any AD? 2) If yes, which type does it belong to and are there any complications (pericardial effusion, thrombosis, etc.)? 3) If not, are there any other aortic lesions (such as aortic aneurysm, atherosclerotic plaque and ulcer, interaortic hematoma, etc.)? In order to achieve the purpose of definite diagnosis and exclusive diagnosis of emergency aortic aneurysm, MRIS can be promoted as a preliminary screening and diagnosis method for clinical aortic aneurysm, and become a fast, simple and non-invasive high-field MR Examination technology in primary hospitals.

## Conclusion

Free-breathing pulse gated non-enhanced rapid MR imaging can obtain images with diagnostic value, and there is no statistical difference in signal-to-noise ratio, contrast noise ratio and blood flow artifact compared with breath-holding electrocardiogram MR imaging, and the examination passing time is significantly lower than that of the latter.

The complications of aortic dissection including internal diaphragm, true and false lumen, involvement of great vascular branches, thrombosis and pericardial effusion were clearly shown, and the rupture was satisfactory. Multi-plane and multi-angle thin-layer scanning were favorable for the display of the rupture, while some complex tears and bidirectional tears were poorly displayed.

Free-breathing pulse gated non-enhanced rapid MR imaging technique is fast, simple and non-invasive, which can be used for the exclusive and definitive diagnosis of aortic dissection.

## Ethics statement

The experiment was approved by the Ethics Committee of the Ezhou Central Hospital Affiliated with Hubei University of Science and Technology (n° 2018EZ08-9), and all patients participating in this study provided written informed consent in accordance with the “Helsinki Declaration”.

## Authors’ contributions

QinWen Yan and CiLai Jiao designed the research study. Si Ma and Lan He performed the research. Lei Wu, QinWen Yan and Gang Hu provided help and advice. Si Ma, Qin Wang and Jun Zhang analyzed the data. MinChao Xiong and QinWen Yan wrote the manuscript. CiLai Jiao and MinChao Xiong reviewed and edited the manuscript. All authors contributed to editorial changes in the manuscript. All authors read and approved the final manuscript.

## Funding

Scientific Research Fund of Hubei Provincial Health Department (WJ2015MA020).

## Declaration of competing interest

The authors declare no potential conflicts of interest with respect to the research, authorship, and/or publication of this article.

## Appendix Supplementary materials

Image, application 1
