# Supplementary material for: Clinical application of pulse-gated non-enhanced rapid magnetic resonance imaging in the definitive diagnosis of aortic dissection
Source: Clinics (Sao Paulo). 2024 Aug 30;79:100467. doi: 10.1016/j.clinsp.2024.100467 (PMC11402381; doi:10.1016/j.clinsp.2024.100467)

**CLINICS-D-23-00696_Supplementary Material**

**Supplementary Figure 1** Coronal bright blood sequence, the tear near the aortic root and the teared internal diaphragm.


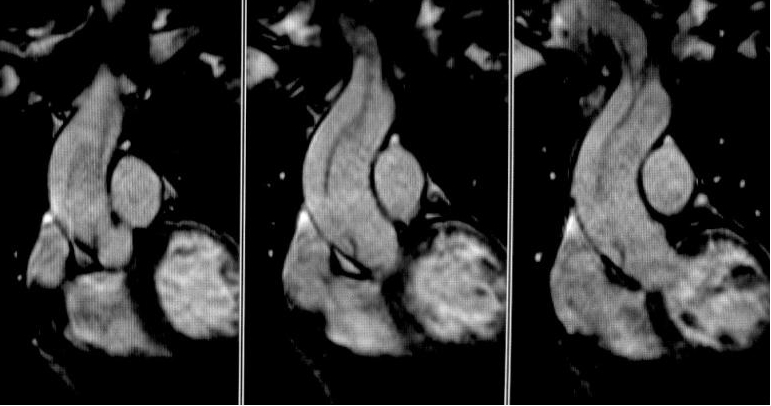


**Supplementary Figure 2** Sagittal bright blood sequence, the tear near the aortic root and the teared internal diaphragm.


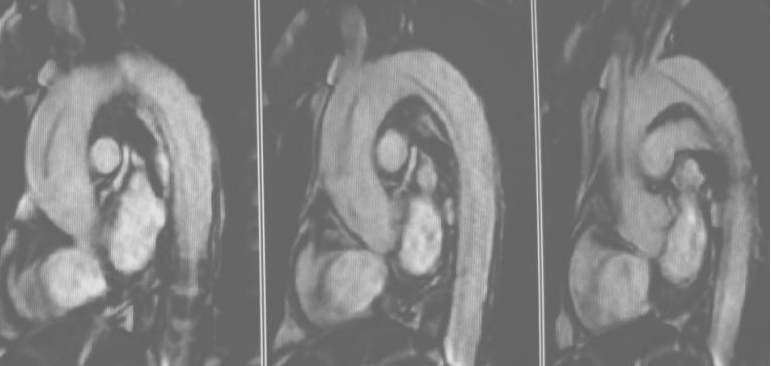


**Supplementary Figure 3** Axial bright blood and black blood sequence, true and false cavity and torn internal diaphragm can be seen. male, 45 years old, Stanford type-a aortic dissection.


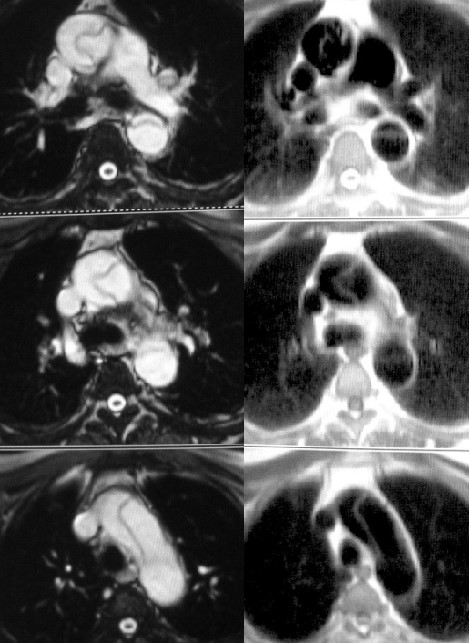


**Supplementary Figure 4** Sagittal bright blood sequence. thrombus formation at the proximal end of descending aorta tear, tearing internal membrane and reverse tear can be seen.


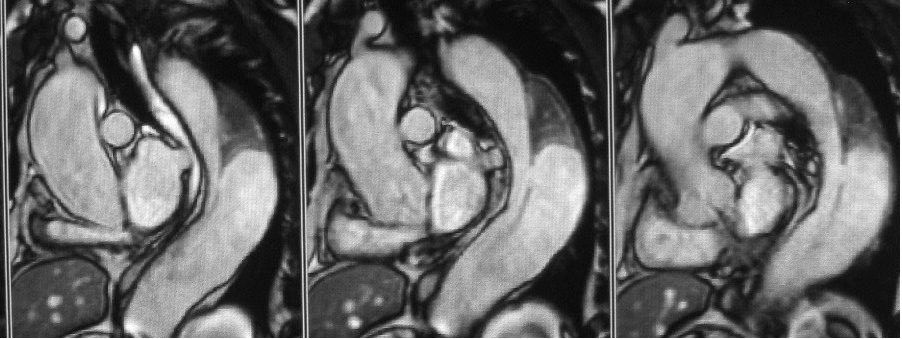


**Supplementary Figure 5** Axial bright blood sequence, the tear of the descending aorta, tearing of the internal diaphragm and thrombosis are visible.


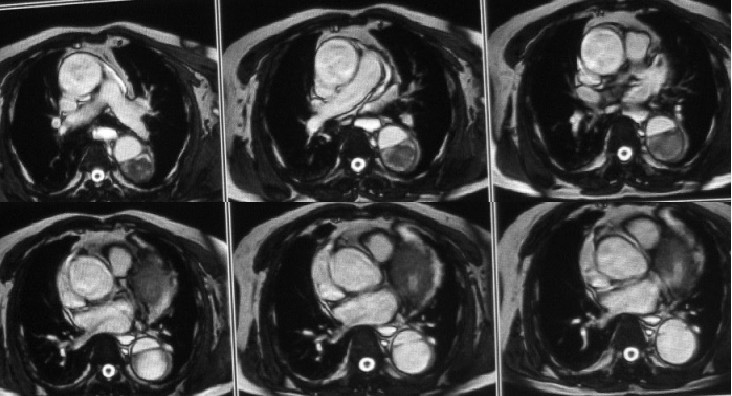


**Supplementary Figure 6** Axial black blood sequence, true and false lumen of descending aorta, torn internal diaphragm and thrombus formation can be seen. 71-year-old male, Stanford type-b aortic dissection.


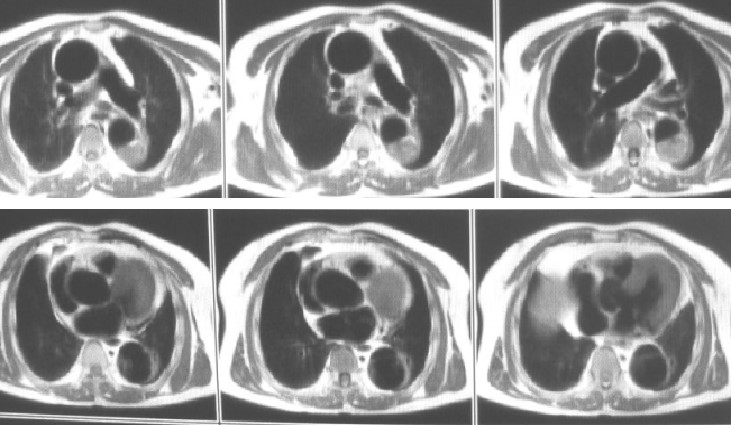


**Supplementary Figure 7** Coronal bright blood sequence, localized dissection of the abdominal aorta can be seen.


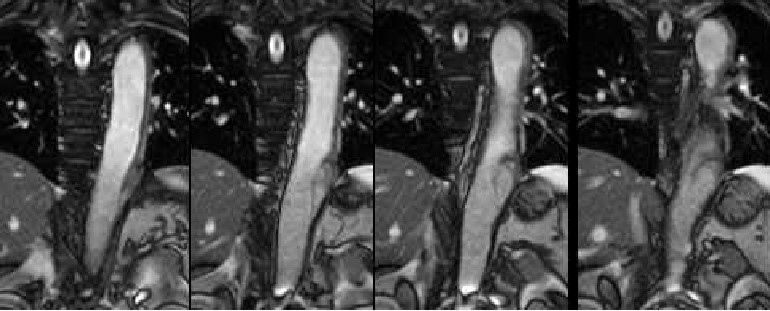


**Supplementary Figure 8** Axial bright blood sequence, crevasse of a localized dissection of the abdominal aorta.


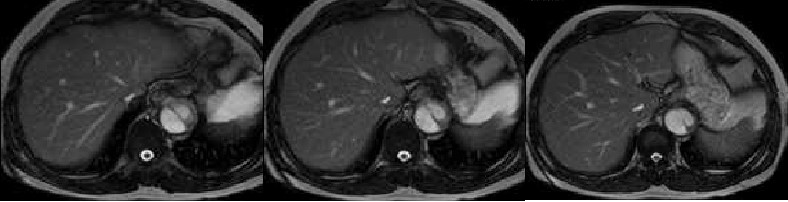


**Supplementary Figure 9** Axial black blood sequence, the true and false lumen of abdominal aortic dissection.


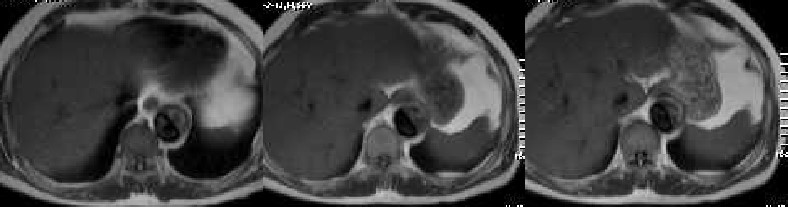

Supplement: Supplementary file 1 [file mmc1.docx]
